# Epigenetic Activation of SOX11 in Lymphoid Neoplasms by Histone Modifications

**DOI:** 10.1371/journal.pone.0021382

**Published:** 2011-06-27

**Authors:** Maria Carmela Vegliante, Cristina Royo, Jara Palomero, Itziar Salaverria, Balazs Balint, Idoia Martín-Guerrero, Xabier Agirre, Amaia Lujambio, Julia Richter, Silvia Xargay-Torrent, Silvia Bea, Luis Hernandez, Anna Enjuanes, María José Calasanz, Andreas Rosenwald, German Ott, José Roman-Gomez, Felipe Prosper, Manel Esteller, Pedro Jares, Reiner Siebert, Elias Campo, José I. Martín-Subero, Virginia Amador

**Affiliations:** 1 Hematopathology Section, Laboratory of Pathology, Hospital Clínic, Institut d'Investigacions Biomèdiques August Pi i Sunyer (IDIBAPS), University of Barcelona, Barcelona, Spain; 2 Institute of Human Genetics, Christian-Albrechts University Kiel and University Hospital Schleswig-Holstein, Campus Kiel, Kiel, Germany; 3 Cancer Epigenetics and Biology Program (PEBC), Bellvitge Biomedical Research Institute (IDIBELL), L'Hospitalet de Llobregat, Barcelona, Spain; 4 Hematology Service and Area of Cell Therapy, Clínica Universidad de Navarra, Foundation for Applied Medical Research, University of Navarra, Pamplona, Spain; 5 Department of Genetics, University of Navarra, Pamplona, Spain; 6 Institute of Pathology, University of Würzburg, Würzburg, Germany; 7 Department of Clinical Pathology, Robert-Bosch-Krankenhaus and Dr. Margarete Fischer-Bosch Institute of Clinical Pharmacology, Stuttgart, Germany; 8 Hematology Department, Reina Sofia Hospital, Cordoba, Spain; 9 Department of Anatomic Pathology, Pharmacology and Microbiology, University of Barcelona, Barcelona, Spain; Instituto Nacional de Câncer, Brazil

## Abstract

Recent studies have shown aberrant expression of SOX11 in various types of aggressive B-cell neoplasms. To elucidate the molecular mechanisms leading to such deregulation, we performed a comprehensive SOX11 gene expression and epigenetic study in stem cells, normal hematopoietic cells and different lymphoid neoplasms. We observed that SOX11 expression is associated with unmethylated DNA and presence of activating histone marks (H3K9/14Ac and H3K4me3) in embryonic stem cells and some aggressive B-cell neoplasms. In contrast, adult stem cells, normal hematopoietic cells and other lymphoid neoplasms do not express SOX11. Such repression was associated with silencing histone marks H3K9me2 and H3K27me3. The *SOX11* promoter of non-malignant cells was consistently unmethylated whereas lymphoid neoplasms with silenced SOX11 tended to acquire DNA hypermethylation. SOX11 silencing in cell lines was reversed by the histone deacetylase inhibitor SAHA but not by the DNA methyltransferase inhibitor AZA. These data indicate that, although DNA hypermethylation of *SOX11* is frequent in lymphoid neoplasms, it seems to be functionally inert, as SOX11 is already silenced in the hematopoietic system. In contrast, the pathogenic role of SOX11 is associated with its de novo expression in some aggressive lymphoid malignancies, which is mediated by a shift from inactivating to activating histone modifications.

## Introduction

The SRY (sex-determining region Y)-box11 (*SOX11*) gene belongs to the SRY-related high-mobility group box gene family of transcription factors, which as a whole controls cell fate and differentiation [1]. SOX11 has been shown to be particularly important for the development of nervous system and adult neurogenesis [2], [3], [4], [5]. SOX11 upregulation has been detected in various types of solid tumors including medulloblastomas, gliomas and epithelial ovarian tumors [6], [7], [8]. Although SOX11 does not seem to play a role in hematopoiesis, its expression has been observed in various aggressive B-cell neoplasms, suggesting that this protein plays a role in the pathogenesis of these tumors. In particular, SOX11 is highly expressed in mantle cell lymphoma (MCL), acute lymphoblastic leukemias (ALL) and in some Burkitt lymphomas (BL) [9], [10], [11], [12], [13]. In contrast, patients with an indolent variant of MCL [14] and other mature B-cell neoplasias such as chronic lymphocytic leukemia (CLL), follicular lymphoma (FL) or diffuse large B-cell lymphoma (DLBCL) do not express SOX11 [9], [10], [12]. Chromosomal changes, like translocations or gene amplifications, constitute one of the main mechanisms leading to deregulated gene expression in lymphomas [15]. In the case of SOX11, chromosomal changes affecting band 2p25.2 (where *SOX11* is located) have not been identified in MCL, BL or ALL [16], [17], [18], [19], [20]. Therefore, other, non-genetic mechanisms should be responsible for its expression pattern in these lymphoid neoplasms. Epigenetic changes like DNA methylation and histone modifications, that regulate gene expression without changing the DNA sequence [21], [22], could be involved in deregulating SOX11 expression in lymphoid neoplasms. In the present study, we have performed a thorough epigenetic characterization of *SOX11*, including DNA methylation and various activating and inactivating histone marks, in several subtypes of non-malignant cells as well as a wide range of lymphoid neoplasia cell lines and primary cases. Our findings show that SOX11 expression is associated with activating histone marks whereas SOX11 repression is associated with inactivating marks with or without the simultaneous presence of DNA methylation.

## Results

### SOX11 gene expression in lymphoid neoplasms by microarrays and qRT-PCR

**Figure 1:**
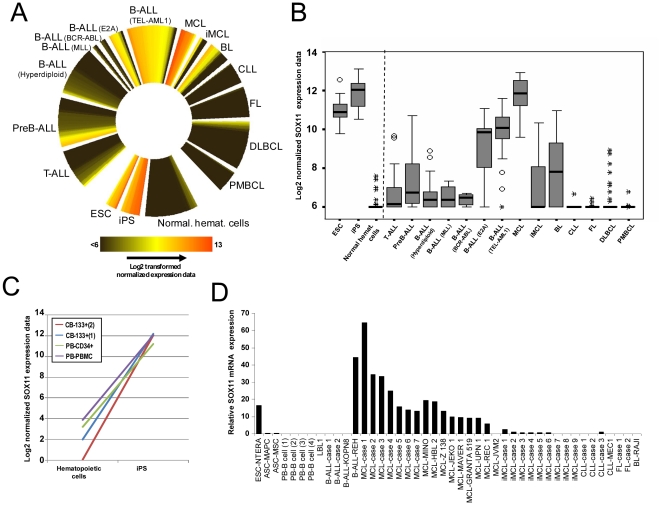
Gene expression analyses of SOX11. (A) Circular heatmap from microarray-data (Table S1) showing the normalized expression levels of 416 samples. SOX11 is consistently expressed in ESC, iPS, MCL as well as B-ALLs with TEL/AML1 fusion or E2A rearrangements. (B) Box-plot summarizing the data shown in panel 1A. (C) Induction of SOX11 in normal hematopoietic cells transformed to iPS by expressing OCT4, SOX2, KLF4 and MYC [23],[24]. (D) Analysis of SOX11 gene expression using qRT-PCR in different lymphoid neoplasm cell lines and primary cases. T-ALL: T-cell acute lymphoblastic leukemia; PreB-ALL: PreB acute lymphoblastic leukemia; B-ALL: B-cell acute lymphoblastic leukemia; MCL: mantle cell lymphoma; iMCL: indolent variant of mantle cell lymphoma; BL: Burkitt lymphoma; CLL: chronic lymphocytic leukemia; FL: follicular lymphoma; DLBCL: diffuse large B-cell lymphoma; PMBCL: primary mediastinal B-cell lymphoma; ESC: embryonic stem cell; iPS: induced pluripotent stem cell; CB: cord blood; PB: peripheral blood; ASC: adult stem cell.

Microarray data confirm that ESCs show high SOX11 expression levels and that SOX11 was not expressed or expressed at very low levels in different hematopoietic cell lineages at various stages of differentiation (Figure 1A–1B, Table S1). Interestingly, upon induction of pluripotent stem cells (iPS) from human hematopoietic cells like CD133+ cord blood cells [23], CD34+ peripheral blood cells or peripheral blood mononuclear cells (PBMC) [24] with different transcription factors (SOX2, OCT4, KLF4 and MYC) , SOX11 is clearly re-expressed (Figure 1C).

In lymphoid neoplasms, SOX11 shows high expression levels in B-ALLs with the TEL-AML1 fusion or E2A rearrangement as well as in the great majority of cases of MCL (Figure 1A–1B). Also, approximately half BL cases express SOX11. In the rest of the neoplasms studied, including additional ALL groups and mature B-cell neoplasms such as CLL, FL, iMCL, DLBCL, primary mediastinal B-cell lymphoma and BL, *SOX11* was either not expressed or expressed at very low levels in a small subset of the cases (Figure 1A–1B).

The qRT-PCR results were in line with the data generated with microarrays. SOX11 was strongly expressed in the embryonic stem cell line NTERA-2, whereas in the two adult stem cells studied (MCS and MAPC) SOX11 was not expressed (Figure 1D). No expression of SOX11 was detected in the four different CD19+ cells purified from healthy blood and the lymphoblastoid B-cell line LBL1. In lymphoid neoplasms, SOX11 was highly expressed in *TEL-AML1*-positive ALL (cell line REH) as well as in all MCLs studied, including eight cell lines and seven primary cases. In contrast, SOX11 was absent in the MCL cell line JVM2, the indolent variants of MCL (nine cases), BCR-ABL-positive B-ALLs (the cell line KOPN8 and two primary cases), CLL (three primary cases), FL (two primary cases) and BL (cell line RAJI) (Figure 1D).

### DNA methylation status of SOX11 by microarrays

**Figure 2:**
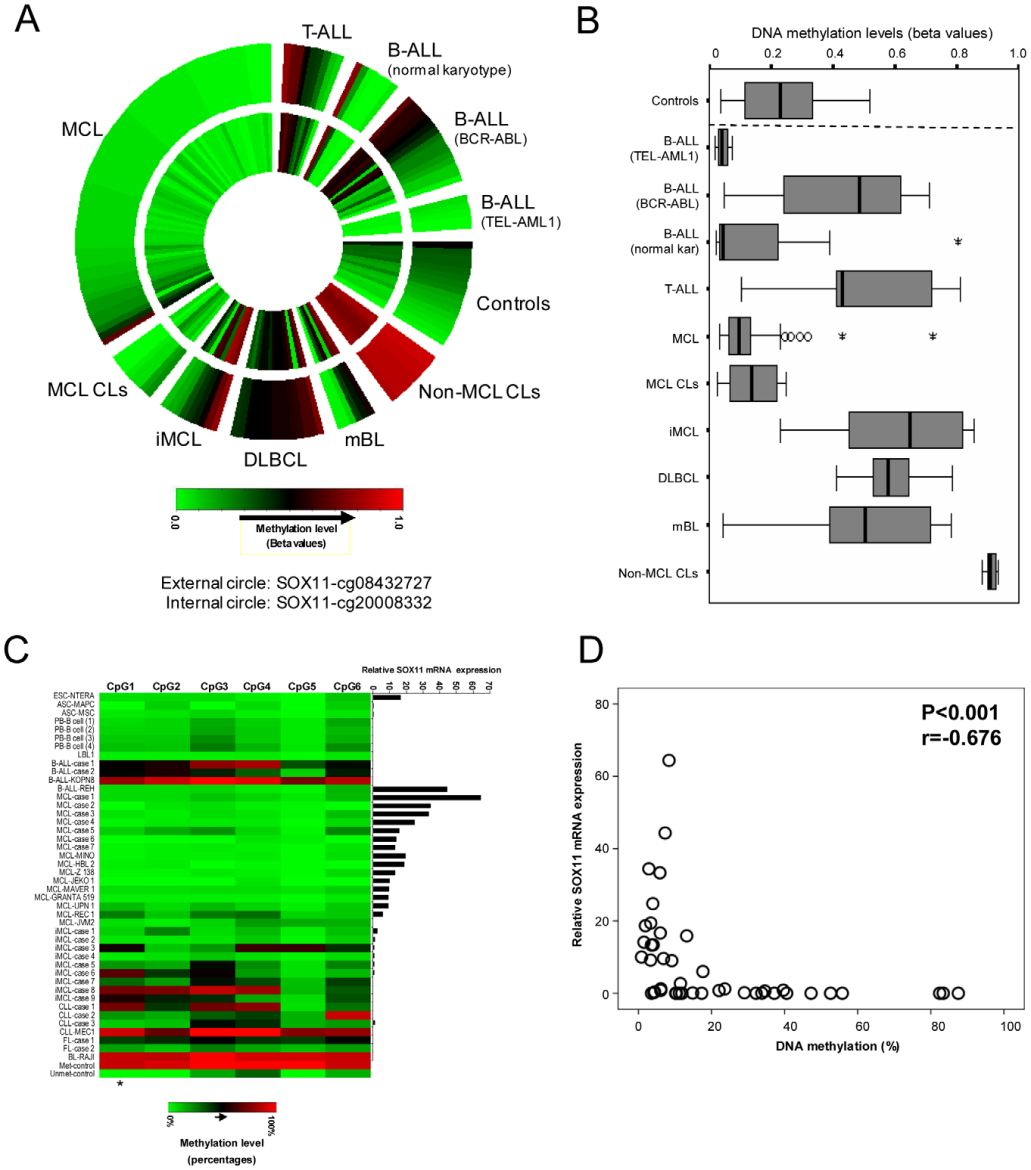
DNA methylation analyses of the promoter region of *SOX11*. (A) Circular heatmap of the two *SOX11*-specific CpGs measured with the 27k Illumina microarray. (B) Box-plot summarizing the data shown in panel 2A. (C) Heatmap of the six *SOX11*-specific CpGs quantified by bisulfite pyrosequencing and SOX11 gene expression analyzed by qRT-PCR. *This CpG is also analyzed by the Infinium array (cg20008332). (D) Scatter plot showing a negative correlation between DNA methylation levels and relative SOX11 mRNA expression (Rho Spearman coefficient = −0.675, p<0.001).

To gain a global insight into the DNA methylation status of *SOX11* in hematological neoplasms and control samples (total n = 159), we used a CpG-specific microarray that includes two CpGs in the 5′ regulatory region of *SOX11* (circular heatmap shown in Figure 2A). In general, both CpGs showed similar DNA methylation values, but as some exceptions were observed, we defined the methylation status of *SOX11* as the maximum of the two values, which was subsequently used to calculate descriptive statistics and the box-plot (Figure 2B). Using this approach, we could determine that various types of normal hematopoietic cells showed low DNA methylation levels (Median/IQR = 0.23/0.22). Cases of ALL were heterogeneous. In those ALLs with the *TEL-AML1* fusion (n = 5) *SOX11* was completely unmethylated (Median/IQR = 0.04/0.04) whereas in other subtypes, like *BCR-ABL* positive (n = 15) or T-ALL (n = 9) *SOX11* exhibited a gradient of DNA methylation values, from unmethylated to methylated cases (Median/IQR of 0.49/0.41 and 0.43/0.40, respectively). MCL primary cases (n = 61) were mostly unmethylated (Median/IQR of 0.10/0.07) and cases of indolent variant of MCL (n = 9) showed a variable degree of DNA methylation (Median/IQR = 0.65/0.44). Aggressive germinal center B-cell lymphomas like DLBCL (n = 14) and molecular BL (mBL, which were defined by transcriptional and genomic profiling) [25] (n = 6) were frequently methylated. DNA methylation values in mBLs showed more heterogeneity (median/IQR = 0.50/0.43) than in DLBCL, in which they were homogeneously methylated (median/IQR = 0.58/0.12) (Figure 2B).

In MCL cell lines (n = 8), *SOX11* was mostly unmethylated (median/IQR = 0.14/0.17) whereas all non-MCL cell lines including T-ALL (n = 1), DLBCL (n = 3), BL (n = 1) and Hodgkin lymphoma (n = 4) were strongly methylated (median/IQR = 0.91/0.03).

These analyses indicate that *SOX11* is mostly unmethylated in normal controls and some types of lymphoid neoplasias like TEL-AML1 positive-ALLs or MCL. In other types of lymphoid neoplasias, however, *SOX11* tends to acquire variable levels of DNA methylation.

### DNA methylation analyses by pyrosequencing and correlation with gene expression

To elucidate whether DNA methylation correlates with SOX11 gene transcription, we quantified the methylation status of six CpGs in the promoter region of *SOX11* using bisulfite pyrosequencing in the same samples used for the expression analysis of SOX11 by qRT-PCR.

The pyrosequencing primer was designed to analyze different CpG sites in the amplified promoter region, including one CpG analyzed by the Infinium array (cg20008332). Twenty six cases (14 primary cases and 12 cell lines) were analyzed by both methods and the DNA methylation values were highly concordant (Rho Spearman coefficient = 0.902, p<0.001, Figure S1). The six CpGs showed similar DNA methylation percentages, indicating the presence of a homogeneous methylation pattern in the *SOX11*-associated CpG island (heatmap shown in Figure 2C).

We defined the methylation status of *SOX11* as the mean of DNA methylation levels among the six CpGs. This single value was subsequently used to study the relationship between DNA methylation and SOX11 gene expression.

In general, a significant inverse correlation between *SOX11* promoter methylation and gene expression was identified (Rho Spearman coefficient = −0.676, p<0.001) (Figure 2D). However, in many samples (embryonic/adult stem cells, normal B cells and some iMCL, some CLL and FL) SOX11 expression was repressed in spite of its unmethylated status. Interestingly, the MCL cell line JVM2 also showed this lack of correlation. This cell line was obtained from a formerly described B-prolymphocytic leukaemia harbouring t(11;14)(q13;q32) translocation cell line. Although JVM2 is considered a MCL cell line, it has a very low number of genetic alterations compared with other MCL cell lines and presents a expression signature similar to indolent MCL, including SOX11 repression. These findings suggest that SOX11 expression does not depend exclusively on the DNA methylation status of the gene and prompted us to study alternative epigenetic mechanisms.

### Detection of histone marks associated with the SOX11 promoter and correlation with gene expression and DNA methylation

To study how the pattern of histone modifications was involved in the regulation of SOX11 expression, we performed quantitative-ChIP assays in samples used for pyrosequencing studies in which at least two million of cells were available.

**Figure 3:**
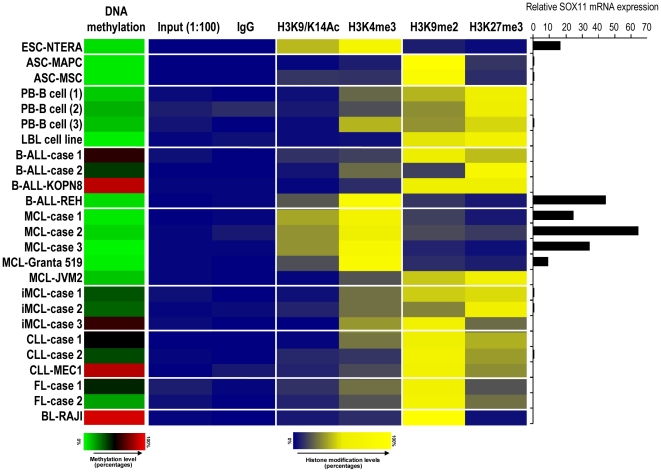
Enrichment of activating and inactivating chromatin marks in *SOX11* promoter and correlation with DNA methylation and gene expression. (Left) Heatmap showing the mean of the six *SOX11*-specific CpGs quantified by bisulfite-pyrosequencing. (Center) Heatmap representing the relative enrichment of H3K4me3 and H3K9/K14Ac as activating chromatin marks and H3K9me2 and H3K27me3 as inactivating chromatin marks in *SOX11* promoter. A rabbit IgG was used as a ChIP negative control. The values are relative to 1∶100 diluted input samples. (Right) Relative SOX11 gene expression analyzed by qRT-PCR.

The relative enrichment of the different marks studied in each sample (H3K4me3, H3Ac, H3K9m2 and H3K27m3) is shown as a heatmap in Figure 3.

We observed that, consistent with expression analyses, *SOX11* promoter in NTERA-2 was enriched for activating chromatin marks (H3K4me3 and H3Ac) and did not show enrichment for repressing marks (H3K9m2 and H3K27m3). On the contrary, in the two types of adult stem cells studied (MCS and MAPC), the four different normal CD19+ cells and the LBL1 cell line, enrichment for repressing histone marks predominates over activating chromatin marks in the *SOX11* promoter, which correlates with the absent expression levels of SOX11 in these samples.

A very similar enrichment pattern as in NTERA-2 was observed in lymphoid neoplasms expressing SOX11. MCLs (GRANTA519 cell line and three primary cases) and the TEL-ALM1 positive ALL (REH cell line) were clearly enriched for activating H3K4me3 and H3Ac chromatin marks. In contrast, samples lacking SOX11 expression, i.e. the MCL cell line JVM2 and iMCL samples (n = 3) as well as the rest of the lymphoid samples (BCR-ABL1-positive ALLs (two primary cases and one cell line (KOPN8)), three CLLs (two primary cases and one cell line (MEC1)), two FL cases and one BL (RAJI)) were enriched for the silencing marks H3K9m2 and H3K27m3 but not for activating marks in *SOX11* promoter (Figure 3).

Analyzing together SOX11 expression, DNA methylation and histone marks in the same cells, our data indicate that SOX11 expression is associated with activating histone marks and absence of DNA methylation. In contrast, lack of SOX11 expression was associated with silencing histone marks, with or without the simultaneous presence of DNA methylation. These results suggest that histone marks, rather than DNA methylation, are the main epigenetic mechanism controlling SOX11 expression.

### SAHA induces SOX11 gene expression in lymphoid cell lines

To confirm the role of histone modifications and DNA methylation in SOX11 gene expression, we investigated gene re-expression, DNA methylation and H3 histone acetylation status after treatment with AZA, SAHA or both. For this study, we used two cell lines with silent SOX11 but different methylation status of *SOX11*, i.e. RAJI (promoter methylated) and JVM2 (promoter unmethylated).

**Figure 4:**
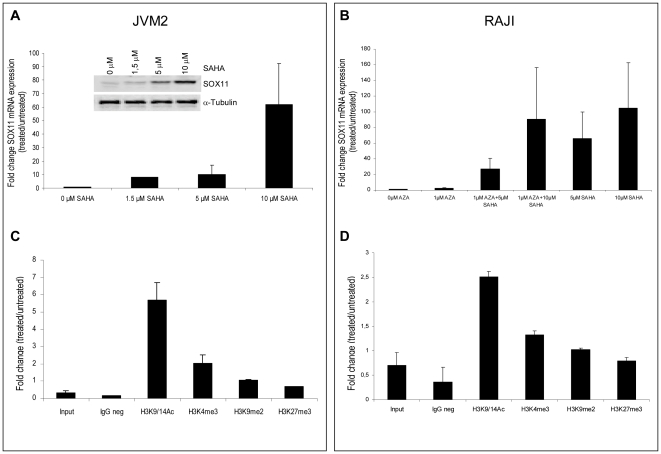
SOX11 gene re-expression and histone modification status analysis after treatments with AZA, SAHA or both in JVM2 and RAJI cell lines. (A) Analysis of relative SOX11 mRNA expression by qRT-PCR and Western blot analysis in JVM2 cells after being treated for 24 h with different concentrations of SAHA (0, 1.5, 5 and 10 µM). (B) Analysis of relative SOX11 gene expression by qRT-PCR in RAJI cells after being treated for 72 h with 1 µM AZA alone or in combination with 5 µM and 10 µM SAHA 24 h concluding the treatment with AZA. For treatment with SAHA alone, 5 µM or 10 µM of SAHA were added to the medium and cultured for 24 h. (C) Enrichment of H3K4me3, H3K9/K14Ac, H3K9me2 and H3K27me3 chromatin marks in the *SOX11* promoter of JVM2 cell line and (D) RAJI cell line treated with SAHA. Values are expressed as relative values of enrichment respect to untreated cells. In JVM2 and RAJI cell lines we observed changes in histone H3 levels after SAHA treatments. To avoid chromatin marks enrichment due to nucleosome increase, levels of H3K4me3, H3K9/K14Ac, H3K9me2 and H3K27me3 chromatin marks had been corrected by the total levels of histone H3 in each cell line.

SAHA treatments, which inhibit histone deacetylases, caused a significant dose-dependent increase in SOX11 mRNA and protein levels in JVM2 (62 fold SOX11 mRNA expression) (Figure 4A) and RAJI (105 fold SOX11 mRNA expression) (Figure 4B). AZA alone, which inhibits DNA methyltransferases, although decreased DNA methylation levels in RAJI (Figure S2), had little influence on SOX11 gene expression in both cell lines. Only a slight increase in RAJI cells was observed (2.4 fold) (Figure 4B).

Histone modifications at the *SOX11* promoter were subsequently measured by quantitative-ChIP analyses after SAHA treatments. In both JVM2 and RAJI cell lines, an increase of H3 acetylation in the *SOX11* promoter was observed in the presence of SAHA (5.7 fold in JVM2 and 2.5 fold in RAJI). The activating H3K4me3 mark was also slightly induced by SAHA treatment (2.05 fold in JVM2 and in 1.3 fold in RAJI cells) (Figure 4C and 4D).

These functional analyses support our previous finding that histone modifications rather than DNA methylation play a predominant role in regulating SOX11 expression.

## Discussion

Several studies have recently demonstrated that SOX11 is up-regulated in various aggressive lymphoid neoplasms [9], [10], [11], [12], [13], [14]. However, the molecular mechanisms leading to such deregulated expression remain unknown. Here, we have performed for the first time a thorough epigenetic characterization of *SOX11* in a wide range of lymphoid malignancies as well as in embryonic/adult stem cells and normal hematopoietic cells.

Our SOX11 expression analyses by microarrays and qRT-PCR extensively confirm and expand previous findings [9], [10], [11], [12], [13], [14]. In non-tumoral cells like ESCs (Figure 1A/1B) and the embryonic cell line NTERA-2 (Figure 1D), SOX11 is highly expressed. However, SOX11 loses its expression in adult progenitor cell types like in MAPCs and MSCs, and all normal hematopoietic cells studied (Figure 1). In contrast, lymphoid malignancies clearly show a differential SOX11 expression among different clinicopathological diseases. In particular, SOX11 is expressed in some subtypes of ALLs (TEL-AML1-positive or with E2A rearrangements), MCLs and part of the BL, but not in any of the other neoplasias analyzed, including the indolent variant of MCL.

As DNA methylation is the most widely studied epigenetic mechanism leading to deregulated gene expression in cancer [21], [22], we initially analyzed the methylation status of *SOX11* promoter by microarrays [26] and bisulfite pyrosequencing [27]. As expected, our findings show that those samples expressing SOX11 are unmethylated. However, adult stem cells and normal hematopoietic cells, although silenced, are consistently unmethylated. In some lymphoid neoplasms without SOX11 expression, this gene acquires variable degrees of DNA methylation. These findings are in line with the DNA methylation of *SOX11* recently reported in CLL, FL and DLBCL [28], [29]. In our series, although SOX11 was silenced in all cases showing methylation, a wide range of samples, from normal cells to lymphoid neoplasms (Figure 2D), were also silenced in spite of an unmethylated status of the *SOX11* promoter. Thus, DNA methylation does not seem to represent a mechanism leading to *de novo* repression in lymphoid neoplasms and in contrast to the conclusion of a recent publication [28], it might not be functionally relevant. The fact that *SOX11* is hypermethylated and silenced in some lymphomas can lead to assume that *SOX11* is a candidate tumor suppressor gene, as recently proposed by Gustavsson and coworkers. However, this assumption must also take into consideration the expression status of this gene in normal lymphoid cells (i.e. expressed in normal cells and repressed in tumor cells). As we here clearly show that *SOX11* is silenced by histone modifications in normal hematopoietic cells, hypermethylation in lymphomas does not modify SOX11 expression levels, and thus, does not seem to have a functional impact.

Combined epigenomic and transcriptomic studies have previously demonstrated that a large proportion of the genes becoming hypermethylated in solid tumors [30] and aggressive B-cell lymphomas [31] are already silenced in their normal cellular counterparts. This finding could be explained by a switch of epigenetic marks between normal cells and tumor samples [32]. As DNA methylation is a more stable repressing mark than histone modifications, it has been hypothesized that tumors reduce their epigenetic plasticity of hypermethylating genes silenced by histone marks in normal cells [32].

In order to gain further insights into SOX11 expression patterns observed in stem cells, normal hematopoietic cells and lymphoid neoplasms, we performed qPCR-ChIP experiments with antibodies against activating and silencing histone modifications. Our data demonstrate that SOX11 expression is associated with histone modifications in all the studied samples. In the embryonic cell line NTERA-2, SOX11 was expressed and its promoter was enriched for the activating marks H3K4me3 and H3K9/K14Ac. Interestingly, an B-ALL with TEL-ALM1 fusion (REH cell line) and all MCLs studied showed the same pattern of activation of *SOX11* as in embryonic stem cells, i.e. enrichment for H3K4me3 and H3K9/K14Ac. This finding is in line with studies proposing that haematological neoplasms acquire chromatin features similar to stem cells [31], [33]. In samples with SOX11 repression, including adult stem cells, normal hematopoietic cells and various lymphoid malignancies, we observed that the *SOX11* promoter was enriched for the silencing marks H3K9me2 and H3K27me3.

To study the causal relationship between SOX11 expression and epigenetic marks, we performed treatments with the AZA and/or SAHA, which inhibit DNA methylation and histone deacetylation enzymes, respectively. SAHA caused the upregulation of SOX11 expression in both JVM2 and RAJI cells, independent of the distinct promoter methylation status in these cells. However, treatment with AZA, although decreased the DNA methylation levels, it did not alter the pattern of histone modifications nor had any effect on the SOX11 gene expression levels in RAJI. Taken together, these findings show that SOX11 expression is associated with activating histone marks whereas SOX11 repression is associated with inactivating marks with or without the simultaneous presence of DNA methylation.

Our data show that the pathogenic effect of SOX11 in lymphoid neoplasias is most likely its aberrant expression associated with activating histone marks in some aggressive B-cell neoplasms. Theoretically, such upregulation could be explained either by a memory of the initial cell from which these neoplasms were originated or by its *de novo* expression. The first hypothesis postulates that SOX11 is expressed in a limited window during B-cell ontogenesis, and that MCLs and some ALLs may derive from such cell type. However, SOX11 is not expressed in any of the normal human hematopoietic cells analyzed, from stem cells to plasma cells (Figure 1A). Additionally, we have performed a more detailed bioinformatic analyses using different mouse hematopoietic cell types derived from the Immunological Genome Project [34]. Using this dataset (GEO accession number GSE15907), SOX11 was not expressed in any of the over 100 hematological cell types studied (Table S2). Therefore, this first hypothesis cannot be supported by experimental data and a *de novo* SOX11 expression caused by a switch from inactivating to activating histone modifications is the most likely explanation. Supporting this view is the fact that reprogramming hematopoietic cells to iPS by inducing expression of OCT4, SOX2, KLF4, and MYC [24], or only OCT4 and SOX2 [23] leads to a *de novo* expression of SOX11 (Figure 1C). Thus, it is likely to hypothesize that genetic or epigenetic changes affecting SOX11 regulators take place in lymphoid neoplasms and result in aberrant *de novo* SOX11 expression. In the case of TEL-AML1-positive B-ALLs it might be that such fusion protein induces SOX11 expression. A recent publication has characterized the transcriptome of cord blood cells after introducing the *TEL-AML1* fusion gene [35]. We extracted SOX11 expression from this study (ArrayExpress identifier E-MEXP-1403) and its expression did not change from wild-type cord blood cells to *TEL-AML1* transfected cells (Table S3). In the case of MCL, CCND1 expression derived from the t(11;14) translocation cannot lead to SOX11 expression, as indolent forms of MCL, that also contain the t(11;14) translocation, do not express SOX11 [18], [19]. Therefore, the upstream mechanisms inducing an open chromatin conformation and subsequent oncogenic upregulation of SOX11 remain unknown.

In conclusion, our data provide a comprehensive characterization of the epigenetic mechanisms leading to SOX11 deregulation in lymphoid neoplasms. As SOX11 is not expressed in normal lymphoid cells, its DNA hypermethylation in some neoplasms without SOX11 expression is most likely functionally inert, and might be associated with reducing epigenetic plasticity in tumor cells [32]. We also show that *de novo* SOX11 expression is associated with aggressive lymphoid malignancies like MCL, some ALL subtypes and a fraction of BL cases, being this effect mediated by a switch between inactivating and activating histone modifications. Furthermore, as SOX11 is strongly expressed in ESCs, our data suggest that SOX11 expression could be associated with the acquisition of stem cell-like chromatin features, as previously proposed [31]. At the mechanistic level, additional studies are required to elucidate which is the functional role of the illegitimate SOX11 expression in lymphoid neoplasms, and which upstream transcription factors and histone modifying enzymes are involved in this phenomenon. At the clinical level, it seems that SOX11 expression confers the cells a more aggressive behaviour, is prognostically important in MCLs [14], and its silencing might represent a suitable strategy for therapeutic intervention.

## Methods

### Cell lines, patient samples and controls

A total of 27 cell lines and 173 primary tumors derived from lymphoid neoplasms were used for gene expression, DNA methylation, histone modification and/or protein analyses. The 27 cell lines included were one B-cell ALL (B-ALL) with *TEL-AML1* fusion (REH), one B-ALL with *BCR-ABL* fusion (KOPN8), one T-cell ALL (T-ALL) (JURKAT), one CLL (MEC1), nine MCL (JVM2, GRANTA519, MINO, JEKO1, Z138, HBL2, UPN1, MAVER1 and HBL2), five DLBCL (VAL, RL, RCK8, LY3 and LY10), two BL (RAJI and DAUDI), as well as seven Hodgkin lymphoma (L1236, L428, KM-H2, HDLM2, L591, L540 and UHO1).

The 173 primary cases studied included 29 B-ALLs (17 with *BCR-ABL* fusion, five with *TEL-AML1* fusion and seven with normal karyotype), nine T-ALLs, seven CLLs, 20 FL, 66 MCLs, 20 DLBCL and 12 BL. All these cases were diagnosed according to the WHO classification [36]. Additionally, we included 10 MCL lacking SOX11 expression. In a previous study we have shown that these MCLs carry the t(11;14), express CCND1, display simple karyotypes, and have an indolent clinical course (stable disease for more than two years without chemotherapy) [14]. These cases will be referred to as indolent MCL (iMCL).

We also studied three types of stem cells: NTERA-2, an embryonal carcinoma cell line widely used as a model of embryonic stem cell (ESC) and two adult stem cells, i.e. one adult mesenchymal stem cell (MCS) and one multipotent adult progenitor cell (MAPC) derived from the bone marrow of healthy individuals.

As normal controls, we used the following samples: two B-cell lines established form normal B-lymphocytes (LBL1 and LBL2), 10 samples of isolated normal CD19+ B cells (seven from peripheral blood and three from tonsils of healthy individuals; all these cells were separated by magnetic-activated cell sorting, Miltenyi Biotech) , one sample of tonsilar germinal center B-cells (CD19+, CD20high, CD38+, separated by subsequent magnetic (Miltenyi Biotech) and fluorescence-activated cell sorting (Becton Dickinson)), three normal lymph nodes, three spleen samples, two bone marrows and one peripheral blood PBLs.

Samples were obtained from the tumor banks of the following institutions: Department of Pathology of the Hospital Clínic (Barcelona, Spain), Institute of Pathology (Würzburg, Germany), Institute of Human Genetics/Pathology Department (Kiel, Germany), Department of Genetics (Pamplona, Spain) or Haematology Department (Cordoba, Spain). The study was approved by the Institutional Review Board of the respective institutions: Department of Pathology of the Hospital clinic, Barcelona, Spain (Hospital Clínic de Barcelona Ethics Institutional Review Board); Institute of Pathology, Wurzburg, Germany (Ethics Committee of the Medical Faculty of the University of Würzburg); Institute of Human Genetics/Pathology Department, Kiel, Germany (Ethics Commission of the Medical Faculty of the Christian-Albrechts-University Kiel); Department of Genetics, Pamplona, Spain (Research Ethics Committee at the University of Navarra); Haematology Department, Cordoba, Spain (Ethics Committee at the University Hospital Reina Sofía).

Written informed consent was obtained from all participants and the ethics committees approved this consent procedure in accordance with the principles of the Declaration of Helsinki.

Table S4 shows a summary of the cell lines, primary cases and controls used for different analyses.

### DNA methylation microarrays

The Infinium Assay from Illumina (San Diego, CA) was used to quantify the DNA methylation status of two CpGs located 1077 (cg08432727) and 610 (cg20008332) base pairs upstream the transcriptional start site of *SOX11*. As this study focuses only on *SOX11*, the rest of the genes studied with the microarray were not considered for the present publication. Array experiments were performed according to the manufacturer's instructions [26].

### Bisulfite pyrosequencing

Genomic DNA was bisulfite converted using the EpiTect Bisulfite Conversion Kit (Qiagen), according to manufacturer's instructions. Bisulfite pyrosequencing was performed according to standard protocols and evaluated with the analysis software Pyro Q-CpG 1.0.9 (Biotage). PCR and primer sequences are shown in Table 1.

**Table 1:** Primers sequences (qRT-PCR, bisulfite pyrosequencing and qPCR-ChIP).

Name	Type	Sequence	Tm (°C)
SOX11_Forward	RT-PCR	CATGTAGACTAATGCAGCCATTGG	60
SOX11_Reverse	RT-PCR	CACGGAGCACGTGTCAATTG	60
SOX11 Probe	RT-PCR	TTTTAACCACGGATAATTG	60
SOX11_FP	RT-PCR	Biotin-TTGGGTAAGAGTTGGAAAATGTTGAA	55
SOX11_RP	RT-PCR	CCTAAACTTAACCCAAAAATCCATTTTAAAC	55
SOX11_seq	RT-PCR	CAAATAATCCACCATATACT	55
SOX11prom_Forward	qPCR-ChIP	GAGAGCTTGGAAGCGGAGA	60
SOX11prom_Forward	qPCR-ChIP	AGTCTGGGTCGCTCTCGTC	60

### SOX11 expression by microarrays and quantitative Real Time-Polymerase Chain Reaction (qRT-PCR)

Previously published stem cell, normal hematopoietic controls and different lymphoid neoplasia raw datasets from HG-U133A and HG-U133 Plus2 Affymetrix gene chips were downloaded from publicly available databases and processed using the R statistical software (http://www.R-project.org) in conjunction with the Bioconductor open source software. Arrays were normalized with the mas5 algorithm and three tags for SOX11 present in both HG-U133A and HG-U133 Plus2 arrays (i.e. 204913_s_at, 204914_s_at and 204915_s_at) were selected. As these three tags showed similar expression levels, they were averaged for further analyses. Table S2 contains a list of the 416 analyzed samples (including GEO identifiers or references) used to generate normalized gene expression intensities.

SOX11 mRNA expression was also investigated by qRT-PCR as described before [12] but with a newly designed primer set and TaqMan® MGB probe for SOX11 using Primer Express® Software Version 2.0 (Applied Biosystems) (Primer set and probe shown in Table 1).

### Quantitative chromatin immunoprecipitation

Chromatin immunoprecipitation (ChIP) experiments were performed with the LowCell ChIP kit (Diagenode; Liege) according to manufacturer's instructions using the following antibodies: H3K9/14Ac, H3K4me3, H3K9me2, H3K27me3 (all four from Diagenode) and H3 (Abcam, ab1791). A rabbit IgG (Diagenode) was used as a negative control.

Immunoprecipitated DNA and 1∶100 diluted input sample were analyzed in triplicate by quantitative real-time PCR analyses using SYBR-Green Master Mix in an ABI 7900 FAST sequence detection system. The primers for the *SOX11* gene promoter region are shown in Table 1.

### Treatment with epigenetic drugs

Lymphoma cell lines were treated for 72 hours with 1 µM 5-aza-2′-deoxycytidine (AZA; Sigma-Aldrich, St. Louis, MO), with drug replacement every 24 hours. For suberoylanilide hydroxamic acid (SAHA; Selleck, Houston, TX) experiments, different concentrations of SAHA (1.5, 5 and 10 µM) were used and cultured during 24 hours. For treatment with both drugs, 10 µM of SAHA was added for the final 24 hours of the 72-hour AZA treatment period. Extension of treatment of cells with AZA (1 to 5 µM) for up to 96 h and higher doses of SAHA caused marked apoptotic effects in the cells.

### Western Blot analysis

Protein extract preparation and Western blot were performed as previously described [37] using a polyclonal rabbit serum against a peptide corresponding to SOX11 specific residues 283-252 (QIKQEPDEEDEEP) generated in our lab (File S1 and Figure S3). A monoclonal antibody anti-α-Tubulin (Oncogene Research, Boston, MA) was used as a loading control.

## Supporting Information

Figure S1 **Scatter plot showing a correlation between DNA methylation percentages of the CpG site 1 quantified by bisulfite pyrosequencing and the values of the CpG analyzed by the Infinium array (cg20008332) (Rho Spearman coefficient = 0.902, p<0.001).**
(TIF)

Figure S2 **Analysis by bisulfite-pyrosequencing of the** ***SOX11*** **promoter de-methylation in RAJI cells after being treated for 72 h with 1 µM AZA alone, in combination with 10 µM SAHA 24 h concluding the treatment with AZA or treated for 24 h with 10 µM of SAHA alone.**
(TIF)

Figure S3 **The specificity of the polyclonal antibody against SOX11 (1159) was verified by western blotting analysis.** HEK293T cells were transfected with vectors encoding HA-SOX4, HA-SOX11 and with the empty vector pcDNA3.1 (CT). Twenty-four hours after transfection, cells were collected and protein extracts were subjected to immunoblotting with antibodies against SOX11 (1159) (left panels) and against HA (Sigma anti-HA; Saint Louis; Missouri) (middle panels), to detect SOX4 and SOX11. The expression levels of SOX11 protein in different MCL cell lines (JVM2, GRANTA519, Z138, JEKO1 and REC1) were detected by using the antibody against SOX11 (1159) (right panels). Differential expression of SOX11 protein in the MCL cell lines, already shown by qRT-PCR, was demonstrated by western blotting. The SOX11-1159 antibody specifically recognized the overexpressed exogenous SOX11 protein as well as endogenous SOX11 protein. The antibody can be used as an important tool for further exploration of the role of SOX11 in tumorigenesis. * Non-specific bands.
(TIF)

Table S1 **Cases studied by Affymetrix gene expression arrays.**
(XLS)

Table S2 **SOX11 mouse-immunological genome project data.**
(XLS)

Table S3 **SOX11 TEL-AML1 transduced cells.**
(XLS)

Table S4 **Cases and analyses.**
(XLS)

File S1 **Supplemental Experimental Procedures.**
(DOC)
