# Supplementary figures and images for: Epigenetic Activation of SOX11 in Lymphoid Neoplasms by Histone Modifications
Source: PLoS One. 2011 Jun 27;6(6):e21382. doi: 10.1371/journal.pone.0021382 (PMC3124503; doi:10.1371/journal.pone.0021382)

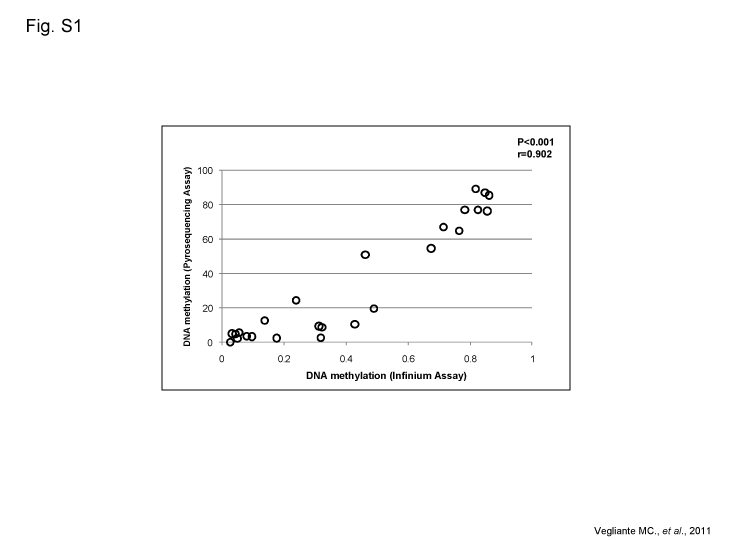

Supplement: Figure S1 — Scatter plot showing a correlation between DNA methylation percentages of the CpG site 1 quantified by bisulfite pyrosequencing and the values of the CpG analyzed by the Infinium array (cg20008332) (Rho Spearman coefficient = 0.902, p<0.001). (TIF) [file pone.0021382.s001.tif]

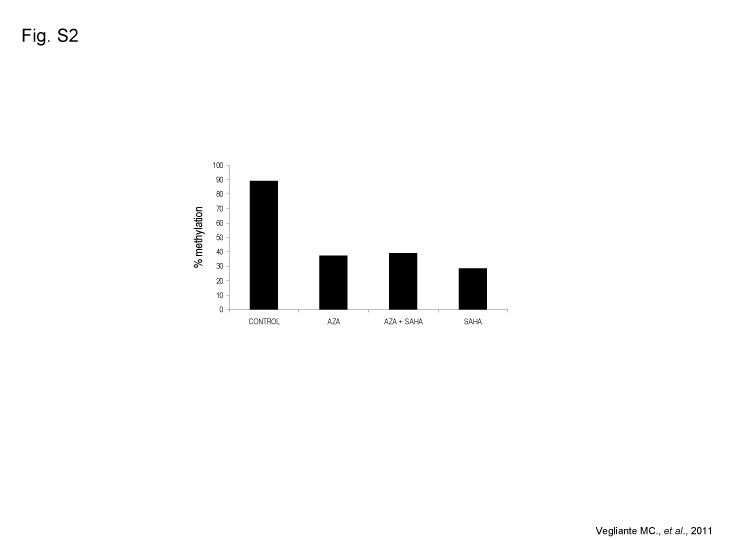

Supplement: Figure S2 — Analysis by bisulfite-pyrosequencing of the SOX11 promoter de-methylation in RAJI cells after being treated for 72 h with 1 µM AZA alone, in combination with 10 µM SAHA 24 h concluding the treatment with AZA or treated for 24 h with 10 µM of SAHA alone. (TIF) [file pone.0021382.s002.tif]

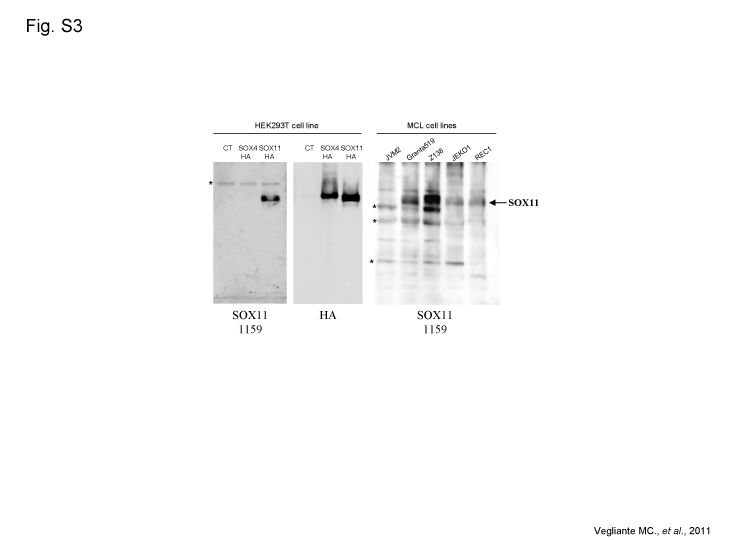

Supplement: Figure S3 — The specificity of the polyclonal antibody against SOX11 (1159) was verified by western blotting analysis. HEK293T cells were transfected with vectors encoding HA-SOX4, HA-SOX11 and with the empty vector pcDNA3.1 (CT). Twenty-four hours after transfection, cells were collected and protein extracts were subjected to immunoblotting with antibodies against SOX11 (1159) (left panels) and against HA (Sigma anti-HA; Saint Louis; Missouri) (middle panels), to detect SOX4 and SOX11. The expression levels of SOX11 protein in different MCL cell lines (JVM2, GRANTA519, Z138, JEKO1 and REC1) were detected by using the antibody against SOX11 (1159) (right panels). Differential expression of SOX11 protein in the MCL cell lines, already shown by qRT-PCR, was demonstrated by western blotting. The SOX11-1159 antibody specifically recognized the overexpressed exogenous SOX11 protein as well as endogenous SOX11 protein. The antibody can be used as an important tool for further exploration of the role of SOX11 in tumorigenesis. * Non-specific bands. (TIF) [file pone.0021382.s003.tif]
