# Supplementary material for: Epigenetic Activation of SOX11 in Lymphoid Neoplasms by Histone Modifications
Source: PLoS One. 2011 Jun 27;6(6):e21382. doi: 10.1371/journal.pone.0021382 (PMC3124503; doi:10.1371/journal.pone.0021382)
Supplement: File S1 — Supplemental Experimental Procedures. (DOC) [file pone.0021382.s008.doc]

**SUPPLEMENTAL EXPERIMENTAL PROCEDURES**

**Cell lines**

Nine well characterized MCL cell lines (1), four B-cell neoplasia cell lines and one embryonal carcinoma cell line were used for gene expression, DNA methylation, histone modification and/or protein analyses. These included HBL2 (kindly provided by Dr M. Dreyling), UPN1 (kindly provided by A. Thurhan), MAVER1 (kindly provided by Dr A. Zamo), Z-138 (kindly provided by Dr E. Ortega-Paino), JEKO1 (CRL-3006, ATCC), JVM-2 (CRL-3002, ATCC), MINO (CRL-3000, ATCC), REC1 (ACC-584, DSMZ) and GRANTA (ACC-342, DSMZ). Other B-cell neoplasia cell lines included: one BL (RAJI, ACC-319, DSMZ), one CLL (MEC1, ACC-497, DSMZ), two B-ALLs (KOPN8, ACC-552, DSMZ; and REH, ACC-22, DSMZ) and the embryonic carcinoma cell line NTERA-2 (ACC-527, DMSZ). These cell lines were cultured in RPMI-1640 containing 10% fetal bovine serum (FBS; Sigma Chemical, St Louis, MO), 2 µM L-glutamine, 100 U/mL penicillin, and 100 µg/mL streptomycin (GIBCO, Grand Island, NY), except for GRANTA-519 that was cultured in DMEM containing 10% FBS, 2 µM L-glutamine, 100 U/mL penicillin, and 100 µg/mL streptomycin and MEC1 that was cultured in IMDM containing 10% FBS, 2 µM L-glutamine, 100 U/mL penicillin, and 100 µg/mL streptomycin. NTERA-2 was cultured in DMEM supplemented with 5% horse serum, 10% FCS, and 1% Pen/Strep (2).

For DNA methylation studies, cell line DNA was obtained from the following sources: L1236 (ACC-530), L-428 (ACC-197), KM-H2 (ACC-8), HDLM-2 (ACC-17), L591 (ACC-602), L540 (ACC-72), U-H01 (ACC-626) and RC-K8 (ACC-561) from the German Collection of Microorganisms and Cell Cultures (DMSZ, Braunschweig, Germany), JURKAT was kindly provided by Dr. M. Brüggemann, DAUDI by Dr. J. Hampe, LY3 by A. Rosenwald, RL by B. Caldwell and VAL and LY10 by R. Shaknovich.

**Plasmids**

*SOX11* and *SOX4* were amplified by PCR using a cDNA library generated from Granta519 cells and JVM2, respectively (PCR primers are shown in Table 2) and Platinum *Pfx*® DNA polymerase (Invitrogen) following the manufacturer’s instructions. The PCR products were inserted into pcDNA3.1 (Invitrogene). The cDNAs were sequenced.

**Table 2. Primers used for cDNA cloning:**

| Name | 5′ - sequence - 3′ |
| --- | --- |
| cSOX11-HA F | cggc**ggatcc***atgtacccatacgatgttccagattacgct*gtgcagcaggcggagagcttg  **BamH1**  *HA-tag* |
| cSox11-HA R | GCCg**CTCGAG**cgcctttcaatatgtgaacac  **XhoI** |
| cSOX4-HA F | cggc**ggatcc***atgtacccatacgatgttccagattacgct*GTGCAGCAAACCAACAATgcc  **bamH1** *HA-tag* |

**Biochemistry**

HA-SOX4 and HA-SOX11 constructs were transfected into HEK293T cells using the Lipofectamine 2000 (Invitrogene), according to manufacturer’s instructions. Forty-eight hours later, cells were collected and lysed in lysis buffer (LB: 50 mM Tris-HCl pH 7.5, 150 µM NaCl, 1 µM EDTA, 50 µM NaF, 0.5% Triton X-100, plus protease inhibitors). Total protein extracts from MCL cell lines (JVM2, GRANTA 519, Z138, JEKO1 and REC1) were collected and lysed in LB. Immunoblotting analyses were performed as previously described (2).

**Antibody**

The rabbit policlonal antibody against SOX11 (SOX11-1159) antibody was generated by injecting rabbits with the peptide QIKQEPDEEDEEP as the antigen, corresponding to amino acids 232-254 of human SOX11. The antibody was then purified from serum using protein A-Sepharose and subsequently by two rounds of affinity chromatography using peptide chromatography (Antibody production facility, “Institut de Biotecnologia i Biomedicina (IBB), UAB, Barcelona). Details on the specificity of this antibody are provided in Figure S3.

**REFERENCES**

1. Salaverria I, Perez-Galan P, Colomer D, Campo E. (2006). Mantle cell lymphoma: from pathology and molecular pathogenesis to new therapeutic perspectives. *Haematologica* 91: 11-16.
2. Aranda P, Agirre X, Ballestar E, Andreu EJ, Román-Gómez J, Prieto I, Martín-Subero JI, Cigudosa JC, Siebert R, Esteller M, Prosper F. (2009). [Epigenetic signatures associated with different levels of differentiation potential in human stem cells.](http://www.ncbi.nlm.nih.gov/pubmed/19915669) *PLoS One* 4:e7809.
3. Amador V, Ge S, Santamaria PG, Guardavaccaro D, Pagano M. (2007). APC/C(Cdc20) controls the ubiquitin-mediated degradation of p21 in prometaphase. *Mol Cell* 27:462-473.

**SUPPLEMENTAL FIGURES:**

**Figure S1.** Scatter plot showing a correlation between DNA methylation percentages of the CpG site 1 quantified by bisulfite pyrosequencing and the values of the CpG analyzed by the Infinium array (cg20008332) (Rho Spearman coefficient=0.902, p<0.001).

**Figure S2.** **Analysis by bisulfite-pyrosequencing of the *SOX11* promoter de-methylation in RAJI cells** after being treated for 72h with 1 μM AZA alone, in combination with 10 µM SAHA 24h concluding the treatment with AZA or treated for 24h with 10 µM of SAHA alone.

**Figure S3**. The specificity of the polyclonal antibody against SOX11 (1159) was verified by western blotting analysis. HEK293T cells were transfected with vectors encoding HA-SOX4, HA-SOX11 and with the empty vector pcDNA3.1 (CT). Twenty-four hours after transfection, cells were collected and protein extracts were subjected to immunoblotting with antibodies against SOX11 (1159) (left panels) and against HA (Sigma anti-HA; Saint Louis; Missouri) (middle panels), to detect SOX4 and SOX11. The expression levels of SOX11 protein in different MCL cell lines (JVM2, GRANTA519, Z138, JEKO1 and REC1) were detected by using the antibody against SOX11 (1159) (right panels). Differential expression of SOX11 protein in the MCL cell lines, already shown by qRT-PCR, was demonstrated by western blotting. The SOX11-1159 antibody specifically recognized the overexpressed exogenous SOX11 protein as well as endogenous SOX11 protein. The antibody can be used as an important tool for further exploration of the role of SOX11 in tumorigenesis. * Non-specific bands.
